# Serological Biomarkers in Early Axial Spondyloarthritis During 24-Months Follow Up (Italian Arm of Space Study)

**DOI:** 10.3389/fmed.2019.00177

**Published:** 2019-08-07

**Authors:** Mariagrazia Lorenzin, Augusta Ortolan, Mara Felicetti, Marta Favero, Stefania Vio, Martina Zaninotto, Pamela Polito, Chiara Cosma, Vanna Scapin, Carmelo Lacognata, Roberta Ramonda

**Affiliations:** ^1^Rheumatology Unit, Department of Medicine–DIMED, University of Padova, Padova, Italy; ^2^Radiology Unit, University of Padova, Padova, Italy; ^3^Medicine of Laboratory, University of Padova, Padova, Italy

**Keywords:** biomarkers, back-pain, early, axial-SpA, MRI, disease-activity

## Abstract

**Objectives:** The study aimed to evaluate biomarkers facilitating early axial-spondyloarthritis (axSpA) diagnosis and disease activity and imaging indices correlated.

**Materials and Methods:** Seventy-five patients with low back pain (LBP) (≥3 months, ≤2 years, onset ≤45 years) participating in the Italian arm of the SpondyloArthritis-Caught-Early (SPACE) study underwent a physical examination, questionnaires, laboratory tests, spine, and sacroiliac joints (SIJ) X-rays and magnetic resonance imaging (MRI) at baseline and during a 24-months follow-up. Two expert rheumatologists formulated axSpA diagnosis and assessed fulfillment of Assessment of SpondyloArthritis International Society (ASAS) criteria. Disease activity and physical functioning were assessed using imaging, clinical, and serological indices. Spine and SIJ MRI and X-rays were scored independently by 2 readers following the *Spondyloarthritis Research Consortium of Canada* (SPARCC), mSASSS, and mNY-criteria. Patients were classified in accordance to ASAS criteria as: 21 patients classified according to axSpA imaging arm; 29 patients classified according to axSpA clinical ± imaging arm; 25 patients not fulfilling ASAS criteria.

**Results:** At baseline biomarker levels were not significantly increased in any of the patient groups. Instead, a significant decrease of all functional and disease activity indices from baseline to 24 months was observed in all the three groups. In the same period, there were no significant variation in the serological markers values within each group. The correlations between IL-17 and IL-23 and clinical and functional indices were not significant. On the other hand, significant correlations were found between IL-22 and Bath Ankylosing Spondylitis Functional Index (BASFI), Bath Ankylosing Spondylitis Patient Global Score (BASG1), Health Assessment Questionnaire (HAQ), Visual Analog Scale (VAS pain); MMP3 and mSASSS; MMP3 and hsCRP.

**Conclusions:** Although not significantly higher in any of the cohorts, IL-22, MMP3, and hsCRP values correlated with some disease activity indices and with mSASSS. Further studies are warranted to confirm these preliminary findings.

## Introduction

Spondyloarthritis (SpA) is a group of debilitating, chronic, rheumatic diseases characterized by overlapping clinical signs and symptoms and a common genetic background (1). Axial spondyloarthritis (axSpA), which mainly affects the spine and the sacroiliac joints (SIJ), has an early onset at a young age and can be further split between non-radiographic axSpA (nr-axSpA) and radiographic axSpA (r-axSpA), the latter also known as ankylosing spondylitis (AS) (2, 3). If undiagnosed and untreated, axSpA may lead to permanent damage and lifelong disability (4, 5). Significant steps forward have been taken in the management of axSpA in clinical practice after the discovery and widespread use of anti-tumor necrosis factor α (TNFα) drugs. It has been demonstrated that axSpA patients respond quickly to anti-TNFα drugs. Indeed, an early diagnosis of these disorders could benefit the patients (1, 4, 5). The Assessment of SpondyloArthritis International Society (ASAS) has established classification criteria to identify patients with early stages of axSpA (2, 3); the imaging arm of the criteria requires the presence of sacroiliitis on magnetic resonance imaging (MRI) or X-rays in addition to one SpA feature for patients with chronic low back pain (LBP) with onset at age ≤45 years (6). Since patients usually display a wide variety of clinical features and there is no standardized laboratory test protocol, the diagnosis of axSpA is rarely straightforward. Nowadays, there is more awareness on the impact of chronic LBP in axSpA patients, and with the recent breakthroughs in genetics research as well as the development of novel treatments with a potentially positive impact in the early disease stages. Recent studies have therefore focused on novel biomarkers that could facilitate early diagnosis and identify those individuals at higher risk for poor prognosis (7, 8). The National Institute of Health Biomarkers and Surrogate Endpoint Working Group has defined biomarker as a “*characteristic that can be objectively measured and evaluated as an indicator of normal biological or pathogenic processes or pharmacological responses to a therapeutic intervention*” (9). Unlike rheumatoid arthritis and other inflammatory arthropathies, there are no specific biomarkers of disease activity in axSpA that are presently being used in clinical practice. Recent studies have focused on the role of some new markers in diagnosing early axSpA, assessing disease activity and identifying patients at higher risk for a worse outcome (7, 8, 10–12). Serum and plasma biomarkers have recently undergone extensive examination and while *Human Leukocyte Antigen* (HLA-B27), the biomarker commonly used in SpA remains relevant, other biomarkers for systemic inflammation such as *C-reactive protein* (CRP) and *Erythrocyte sedimentation rate* (ESR), usually used in clinical practice, are often unable to assess disease activity in axSpA (13–16). As early treatment can reduce the disease burden in axSpA patients and disease-related costs, uncovering biomarkers that can help early diagnosis of axSpA has become an urgent undertaking.

## Materials and Methods

### Patients

The SpondyloArthritis-Caught-Early (SPACE) study is an on-going observational cohort study that was originally launched at the Leiden University Medical Center (LUMC, The Netherlands) in January 2009. Patients who are at least 16 years old suffering with inflammatory LBP (≥3 months, ≤2 years, onset <45 years) of unknown origin are included in this cohort. Participating centers include rheumatology outpatient clinics in the Netherlands (Amsterdam, Gouda, Leiden), Norway (Oslo), Sweden (multiple sites), and Italy (Padua). Specifically, in March 2012 the SpA study group of the Rheumatology Unit at the University of Padua opened an Italian branch of the SPACE cohort. In the present study, only patients from our center have been included. Approval by local medical ethics committees (Medical Ethics Committee, Leiden University Medical Center [approval no. P08.105]; and Azienda Ospedaliera di Padova [approval no. 2438P]) was obtained. Informed consent was obtained from the patients prior to inclusion. Patients qualifying for the study (2, 3) were assessed following a specific protocol including physical examination, laboratory tests, SIJ/spine plain radiographs and MRI, questionnaires' regarding disease activity and functional state. The SpA features considered for diagnosis in accordance with the ASAS criteria (2, 3) were radiographic sacroiliitis (Modified New York = mNY criteria = bilateral grade ≥2 or unilateral grade ≥3), sacroiliitis on MRI (presence of ≥2 inflammatory lesions highly suggestive of axSpA on a single slice or one lesion on ≥2 consecutive slices), HLA-B27 positivity, positive family history for SpA, inflammatory back pain (IBP), psoriasis, peripheral arthritis, dactylitis, heel enthesitis, uveitis, inflammatory bowel disease (IBD), good response to *non-steroidal anti-inflammatory drugs* (NSAIDs) and elevated CRP or ESR. After this, a diagnosis of axSpA or non-axSpA was performed by two expert rheumatologists (RR and ML); the agreement between the two clinics was good (k 0.75); only axSpA or suspected axSpA patients were considered eligible for this study. All clinical, imaging, disease activity, and serological indices were assessed, following a standardized protocol, at baseline (T0), at 6 months (T6), at 12 months (T12), and 24 months (T24). At T0, all the patients were being treated with only NSAIDs and no *conventional synthetic disease modifying anti-rheumatic drugs* (csDMARDs) or biologics. Afterwards, patients were treated according to best clinical practice, with no limitations on pharmacological treatments, physical therapies or other treatments. The protocols for imaging (MRI and plain radiograph) and for patient assessment, including metrology, and clinimetric indices (Bath Ankylosing Spondylitis Metrology Index (BASMI) and Maastricht Ankylosing enthesitis Spondilities Score (MASES), functional, and disease activity indices [Bath Ankylosing Spondylitis Disease Activity Index (BASDAI); Bath Ankylosing Spondylitis Functional Index (BASFI); Visual Analog Scale (VAS pain); VAS night pain, VAS disease activity; Bath Ankylosing Spondylitis Patient Global Score (BASG1); BASG2; Health Assessment Questionnaire (HAQ); Ankylosing Spondylitis disease activity score (ASDAS)], have been described elsewhere by Lorenzin et al. (17). Lateral view radiographs of the cervical and lumbar spine and anterior-posterior view radiographs of the pelvis were taken. The images were obtained with a Philips vertical bucky, with a focus-film distance of 140 cm, film size of 18 × 43 cm. SIJ and spinal MRIs were performed at baseline using a 1.5T scanner Magnetom Harmony, Siemens AG Medical Solutions, Munich, with phased-array surphace coil, acquiring T1-weighted turbo spin echo (T1TSE; TR 550/TE 10) and short-tau inversion recovery (STIR; TR 2500/TE60) sequences. The coronal oblique and sagittal views of the SIJ and spine were in 4 mm slice thicknesses. As mentioned earlier, MRI images were analyzed in accordance with the ASAS/OMERACT criteria (6, 18) and the *Spondyloarthritis Research Consortium of Canada* (SPARCC) score (19, 20), while spine X-Rays and SIJ X-Rays were scored in accordance with the *Stoke Ankylosing Spondylitis Spinal Score (SASSS) system* modified by Creemers (mSASSS) (21) and mNY criteria (22). The patients' X-rays and MRI images were scored independently by two expert readers (SV and VS), and the patients were classified, in accordance with the ASAS criteria for axSpA (2, 3), into three groups: those fulfilling only the imaging arm of ASAS axSpA criteria (*axSpA imaging arm*), those fulfilling the clinical arm of ASAS axSpA criteria in presence/absence also of imaging arm (*axSpA clinical* ± *imaging arm)* and those not completely fulfilling ASAS axSpA criteria (*not full ASAS axSpA*). The two readers scored positive, the image was scored accordingly. All readers were blinded for clinical and laboratory data, and for the results of the other imaging methods. The temporal sequence of the images was unblinded to radiologists for the scoring. The mean scores were calculated using those of both of the readers. The inter-observer reliability was, respectively, good to moderate (kappa 0.73 for inflammatory lesions and 0.60 for structural lesions on spine MRI) and good to moderate (kappa 0.78 for inflammatory lesions and 0.61 for structural lesions on SIJ MRI). An adjudicator (CLC) was introduced when readers disagreed on a positive SIJ MRI or positive spine MRI. If the primary readers agreed on a positive (or negative) SIJ MRI, the mean SPARCC scores were calculated based on the scores of these primary readers. In cases of disagreement, the mean scores were based on the consensus scores of the adjudicator and 1 primary reader. A similar process was followed for calculating the mean SPARCC scores in the spine MRI. The inter-observer reliability for all X rays images was good (kappa 0.79 for spine radiological lesions and kappa 0.77 for SIJ radiological lesions). The adjudicator (CLC) was introduced in case of disagreement between the readers regarding the presence/absence of spine or SIJ radiological lesions on X-rays. The intra-observer reliability was good for all spine and SIJ images on X-rays and MRI (respectively kappa 0.79 for spine and 0.83 for SIJ).

### Serological Evaluation

Erythrocyte-sedimentation rate [normal range 0–15 mm/h] and CRP [normal range 0–6 mg/L] values were determined. Serum ultrasensitive CRP (hs-CRP), (Research & Diagnostic Systems, Inc., expressed in mg/L, with a lower limit of detection of 0 mg/L); *matrix metalloproteinase* (MMP3), (Quantikine MMP3 R&D Systems Europe, expressed in ng/mL, with a lower limit of detection of 0 ng/mL); *interleukin* IL-22, IL-17, and IL-23 (R&D Systems Europe, expressed in pg/mL, with lower limit of detection, respectively of 5 pg/mL, 17 pg/ml, of 20 pg/mL) were assessed using an enzyme-linked immunosorbent assay (ELISA). Serum for the assays was separated by centrifugation at 3,000 rpm for 10 min and stored at −80°C. All blood samples were analyzed twice using the same method.

### Statistical Analysis

Cohen's Kappa was used for intra and inter-observer reliability. Since the examined variables were not normally distributed, differences among T0, T6, T12, and T24 indices were evaluated using the non-parametric Kruskal-Wallis test (one-way ANOVA for ranks) for repeated measurements followed by the Dunn's multiple Comparison test. These tests were therefore used to compare the clinical (BASMI, MASES), functional (BASFI, HAQ, BASG1, BASG2, VAS pain, VAS night pain, VAS disease activity), serological (ESR, CRP, hs-CRP, MMP3, IL-22, IL-17, IL-23), and disease activity indices (BASDAI, ASDAS) at T0, T6, T12, and T24. The same method was used to compare the imaging scores (mSASSS, score SIJ mNY, SPARCC SIJ, and SPARCC spine) at T0, T12, and T24 in all patients and among the three cohorts (*axSpA imaging arm, axSpA clinical* ± *imaging arm and not full ASAS axSpA*). The Spearman test was used to analyze the correlations between clinical, serological, disease activity, and imaging score indices. A *p* value < 0.05 was considered significant.

## Results

Seventy-five axSpA patients were enrolled. According to the ASAS criteria, 21 (28%) patients were classified as *axSpA imaging arm*, 29 (38.7%) patients as *axSpA clinical* ± *imaging arm* and 25 (33.3%) patients did *not fulfill ASAS criteria*. MRI SIJ inflammatory lesions defined as bone marrow edema were present in 46 (61.3%) patients, respectively in 21 (100%) patients in the *axSpA imaging arm* and in 25 (86.2%) patients in *axSpA clinical* ± *imaging arm*.

**Figure 1:**
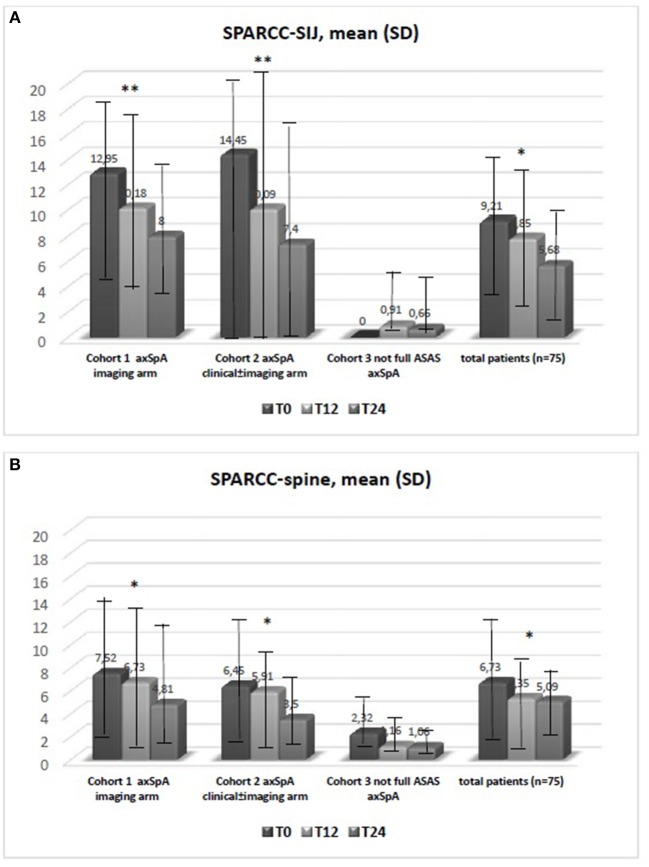
**(A,B)** Data are expressed as mean SD. The test kruskal wallis repeated measures test and Dunn's multiple comparison test were used. *p < 0.01 vs. T0, **p < 0.001 vs. T0. Spondyloarthritis Research Consortium of Canada (SPARCC); sacroiliac joint (SIJ).

The average age at LBP onset was 28.51 ± 8.05 years, 45.3% were male, 38.7% were HLA-B27+. Thirty nine (52%) patients presented an exclusive axial involvement, while−36 (48%) of patients also had peripheral involvement. A high prevalence of psoriasis and heel enthesitis was observed (33.3 and 72%, respectively). Other characteristics of the patients, including the typical aspects for SpA, are reported in Table 1. As expected, significant correlations in the overall population were found between some clinical indices (BASFI, BASG1, BASG2, VAS) and between clinical and disease activity indices (BASDAI, ASDAS) (data not shown). The correlations between the clinical indices, MMP-3 and IL-22 and between ESR, hsCRP, and clinical indices are outlined in Tables 2, 3 and have been previously described and published elsewhere (16). Patients' clinical and disease activity indices, biomarkers, and imaging scores at T0 are outlined in Table 4. According to the Kruskal Wallis test, there were significant differences in the 3 cohorts as it relates to the prevalence of radiographic sacroiliitis, active sacroiliitis on MRI and the SPARCC SIJ scores (Table 4). There were no differences in the serological markers (ILs, MMP3, and hsCRP) at T0 in the three groups. Among all 75 patients, 90.7% were evaluated at T6; 78.7% at T12; 72% at T24. The clinical (MASES and BASMI), disease activity (BASDAI, ASDAS), functional (HAQ, BASFI, VAS pain, VAS pain night, VAS disease activity, BASG1, BASG2), and serological indices (ESR, CRP, hs-CRP, MMP3, IL-22, IL-17, IL-23) were analyzed and measured over time at T0, T6, T12, and T24 and reported in Tables 5, **6**. Considering the whole population, a significant decrease of the following parameters from T0 to T24 was highlighted: MASES (*p* = 0.008), BASG1 (*p* = 0.02), BASG2 (*p* < 0.0001), HAQ (*p* = 0.0002), VAS pain (*p* = 0.01), VAS pain night (*p* = 0.04), VAS disease activity (*p* = 0.05), BASFI (*p* = 0.02), BASDAI (*p* < 0.0001), ASDAS (*p* < 0.0001). Conversely, BASMI, ESR, CRP, and hsCRP as well as MMP-3 and ILs did not decrease significantly. Considering the patients subdivided in the 3 cohorts, a slight downward trend for all functional indices (HAQ, BASFI, BASG1, BASG2, VAS pain, VAS night pain, and VAS disease activity) and disease activity indices (BASDAI, ASDAS) was observed, which in some cases was statistically significant (see Tables 5, 6), but there was no more markedly significant decrease of these indices in one cohort compared to the other two. A significant difference as regards SPARCC SIJ and SPARCC spine scores was also observed in *axSpA imaging arm* cohort and *axSpA clinical*±*imaging arm* cohort with tendency of decreasing values from T0 to T24 (Figures 1A,B).

**Table 1:** Baseline characteristics of LBP patients (*n* = 75).

Age of onset LBP, mean (±SD)	28.51 (±8.05)
Male, *n* (%)	34 (45.3%)
LBP duration (months), mean (±SD)	13.37 (±6.14)
Only axial involvement, *n* (%)	39 (52%)
Axial and peripheral involvment, *n* (%)	36 (48%)
HLA-B27 positive, *n* (%)	29 (38.7%)
Positive family history of SpA, *n* (%)	35 (46.7%)
IBP, *n* (%)	75 (100%)
Peripheral arthritis, *n* (%)	34 (45.3%)
Psoriasis, *n* (%)	25 (33.3%)
Dactylitis, *n* (%)	15 (20%)
Heel enthesitis, *n* (%)	54 (72%)
Uveitis, *n* (%)	7 (9.3%)
IBD, *n* (%)	9 (12%)
Preceding infections, *n* (%)^†^	4 (5.3%)
Good response to NSAIDs, *n* (%)	73 (97.3%)
Elevated CRP/ESR, *n* (%)	42 (56%)
Cervical pain, *n* (%)	28 (37.3%)
Thoracic pain, *n* (%)	42 (56%)
Buttock pain, *n* (%)	48 (64%)
Alternating buttock pain, *n* (%)	37 (49.3%)
Morning stiffness, *n* (%)	57 (76%)
Night pain, *n* (%)	71 (94.7%)
Sacroiliitis MRI ^*^, *n* (%)	46 (61.3%)
Sacroiliitis x-ray ^**^, *n* (%)	25 (33.3%)
Weight (kg), mean (±SD)	70.22 (16.15)
Height (cm), mean (±SD)	170.6 (8.67)

*HLA-B27, Human Leukocyte Antigen; LBP, low back pain; IBP, Inflammatory Back Pain; IBD Inflammatory Bowel Disease; CRP, C-reactive protein; ESR, erythrocyte sedimentation rate; MRI, Magnetic Resonance Imaging; SpA, Spondyloarthritis; NSAID, non-steroidal anti-inflammatory drugs*.

* *Sacroiliitis on MRI according ASAS/EULAR criteria*.

** *Sacroiliitis on X-Rays according modified New York criteria (0–4)*.

† *Balanitis, Urethritis or Cervicitis*.

**Table 2:** Correlations between MMP3, IL-22 and clinical indices and imaging scores.

	**MMP3**	**IL-22**
ESR	0.26 (0.041)	ns
hsCRP	0.28 (0.029)	ns
mSASSS	0.31 (0.005)	ns
SPARCC SIJ	ns	0.043 (0.031)
BASMI	ns	0.28 (0.040)
BASFI	ns	0.35 (0.008)
BASG1	ns	0.34 (0.008)
HAQ	ns	0.27 (0.040)
VAS pain	ns	0.26 (0.013)

*P was calculated according to Spearman's correlation test. Data are expressed as correlation coefficient (p value)*.

**Table 3:** Correlations between hsCRP, ESR and clinical indices and imaging scores.

	**hsCRP**	**ESR**
BASG1	ns	0.27 (0.028)
BASG2	ns	0.26 (0.041)
ASDAS	ns	0.29 (0.025)
SPARCC SIJ	0.35 (0.047)	Ns
mSASSS	ns	−0.12 (0.038)

*P was calculated according to Spearman's correlation test. Data are expressed as correlation coefficient (p value)*.

**Table 4:** Serological, clinical, disease activity indices and imaging score at T0 in the whole group of patients (*n* = 75) and in the three cohorts (axSpA imaging arm, axSpA clinical ± imaging arm, not full ASAS axSpA).

**Serological, clinical, disease activity, and imaging score indices**	**Cohort 1 axSpA imaging arm, *n* = 21**	**Cohort 2 axSpA clinical ± imaging arm, *n* = 29**	**Cohort 3 not full ASAS axSpA, *n* = 25**	***p*^§^**	**Total = 75 pts**
ESR (mm/h), mean (SD)	17.52 (12.98)	15.14 (11.76)	21.68 (21.19)	ns	17.99 (15.85)
CRP (mg/L), mean (SD)	4.81 (3.61)	3.17 (3.32)	4.24 (3.28)	ns	3.98 (3.42)
hs-CRP (mg/L), mean (SD)	2.54 (2.79)	1.56 (1.71)	2.76 (3.76)	ns	2.11 (2.50)
MMP3 (ng/L), mean (SD)	3.04 (2.69)	3.51 (3.12)	2.47 (2.68)	ns	3.01 (2.80)
IL-22 (pg/mL), mean (SD)	6.44 (3.52)	5.91 (2.45)	9.83 (8.54)	ns	7.58 (11.69)
IL-17 (pg/mL), mean (SD)	3 (3.12)	3 (3.11)	3 (3.97)	ns	3 (4.11)
IL-23 (pg/mL), mean (SD)	29.3 (31.2)	21.87 (5.21)	20.69 (21.56)	ns	21.53 (60.56)
HLA-B27, *n* (%)	0 (0)	29 (38.7%)	0 (0)	ns	29 (38.7)
BASMI, mean (SD)	1 (1.18)	0.76 (1.02)	0.92 (1.08)	ns	0.88 (1.08)
MASES, mean (SD)	3.33 (2.65)	3.41 (2.35)	3.56 (2.47)	ns	3.44 (2.45)
BASFI, mean (SD)	17.43 (20.21)	13.68 (15.14)	22.44 (25.75)	ns	17.65 (20.64)
HAQ, media (DS)	0.34 (0.53)	0.30 (0.38)	0.52 (0.55)	ns	0.39 (0.49)
BASG1, media (DS)	3.43 (2.79)	3.52 (2.77)	4.64 (3.35)	ns	3.87 (2.99)
BASG2, media (DS)	5.71 (3.02)	5.10 (2.77)	5.28 (2.94)	ns	5.33 (2.87)
VAS pain, mean (SD)	3.48 (2.82)	4.10 (2.97)	5.08 (3.23)	ns	4.25 (3.05)
VAS disease activity, mean (SD)	3.81 (3.03)	4.07 (3.15)	5.04 (3.35)	ns	4.32 (2.18)
VAS pain night, mean (SD)	3.24 (3.33)	4.17 (3.57)	4.20 (3.33)	ns	3.92 (3.40)
BASDAI, mean (SD)	39.50 (25.99)	44.27 (25.03)	53.48 (24.97)	ns	46.01 (25.07)
ASDAS, mean (SD)	2.29 (0.86)	2.61 (0.56)	2.66 (0.88)	ns	2.54 (0.77)
Sacroiliitis x-ray ^**^, *n* (%)	11 (52.4)	14 (48.3)	0 (0)	<0.001	25 (33.3)
Sacroiilitis MRI ^*^, *n* (%)	21 (100)	25 (86.2)	0 (0)	<0.001	46 (61.3)
mSASSS, mean (SD)	3.57 (4.25)	2.14 (2.84)	2.48 (3.82)	ns	2.65 (3.61)
score SIJ mNY^*^, mean (SD)	0.61 (0.67)	0.79 (0.98)	0 (0)	<0.01	0.55 (0.78)
SPARCC spine, mean (SD)	6.52 (14.28)	6.45 (10.98)	2.32 (4.31)	ns	5.09 (10.52)
SPARCC SIJ, mean (SD)	12.95 (8.23)	14.45 (15.11)	0 (0)	<0.001	9.21 (12.28)

*ESR, erytrocyte sedimentation rate; CRP, C-reactive protein; hs-CRP, high sensitive C-reactive protein; MMP3 matrix-metallo-proteinase 3; IL, Interleukine; HLA-B27, Human Leukocyte Antigen; BASMI, Bath Ankylosing Score Metrology Index; MASES, Maastricht Ankylosing Spondilities Enthesitis Score; BASFI: Bath Ankylosing Spondylitis Functional Index, HAQ: Health Assessment Questionnaire; BASG1, Bath Ankylosing Spondylitis Patient Global Score 1; BASG2, Bath Ankylosing Spondylitis Patient Global Score 2;BASDAI: Bath Ankylosing Spondylitis Disease Activity Index, VAS, Visual Analog Scale; ASDAS, Ankylosing Spondylitis Disease Activity Score; SPARCC, Spondyloarthritis Research Consortium of Canada; mSASSS, modified Stoke Ankylosing Spondylitis Spine Score; SIJ, sacroiliac joints*.

* *Sacroiilitis MRI according ASAS/EULAR*.

** *Sacroiliitis x-ray according New York criteria*.

*^§^ Anova (Kruskal Wallis) a t0: p < 0.05. SD, deviation standard*.

**Table 5:** Values of clinical and functional indices from T0 to T24 in the whole group of patients (*n* = 75) and in three cohorts (axSpA imaging arm, axSpA clinical ± imaging arm, not full ASAS axSpA).

		**BASMI**	**MASES**	**BASFI**	**HAQ**	**BASG1**	**BASG2**	**VAS pain**	**VAS dis act**	**VAS pain N**
axSpA imaging arm	T0	1 (1.18)	3.33 (2.65)	17.43 (20.21)	0.34 (0.53)	3.43 (2.79)	5.71 (3.02)	3.48 (2.82)	3.81 (3.03)	3.24 (3.33)
	T6	0.80 (1.15)	3.30 (2.85)	17.95 (22.52)	0.21 (036)	3.15 (2.87)	4.80 (2.48)	3.40 (2.72)	3.35(2.91)	2.90 (2.92)
	T12	0.39 (0.78)	3.06 (2.98)	12.67 (12.82)	0.15 (0.31)	2.01(2.03)	3.71 (2.44)	3.47 (2.61)	3.18 (2.74)	2.06 (2.34)
	T24	0.44 (0.63)	2.06 (2.11)^*^	9.25 (8.12)^**^	0.14 (0.38)^**^	2.29 (2.23)^*^	2.71 (2.64)^**^	1.75 (1.77)^*^	1.81 (1.71)^*^	1.56 (1.93)^*^
axSpA clinical ± imaging arm	T0	0.76 (1.02)	3.41 (2.35)	13.680 (15.14)	0.30 (0.38)	3.52 (2.77)	5.10 (2.77)	4.10 (2.97)	4.07 (3.15)	4.17 (3,57)
	T6	0.54 (0.86)	2.58 (2.39)	15.85 (20.65)	0.21 (0.37)	2.73(2.54)	3.84 (2.31)	3.08 (2.64)	3.19 (2.80)	2.62 (2.70)
	T12	0.43 (0.79)	2.39 (2.90)	11.04 (15.63)	0.10 (0.25)	2.67 (2.33)	3.33 (2.12)	2.41 (2.04)^*^	2.92 (2.22)	2.25 (2.26)^*^
	T24	0.50 (0.86)	2.09 (2.37)^*^	10.27 (14.29)^*^	0.10 (0.23)^*^	1.50 (1.87)^**^	1.91 (1.98)^**^	2.23 (2.20)^*^	2.18 (2.32)^*^	2.36 (2.32)^*^
not full ASAS axSpA	T0	0.92 (1.08)	3.56 (2.47)	22.44 (25.75)	0.52 (0.55)	4.64 (3.35)	5.28 (2.94)	5.08 (3.23)	5.04 (3.35)	4.20 (3.33)
	T6	0.96 (1.22)	3.43 (2.59)	17.52 (21.71)	0.34 (0.41)	4.26 (3.01)	4.82 (3.52)	4.52 (3.36)	4.30 (3.21)	3.48 (3.17)
	T12	0.65 (0.81)	2.40 (2.14)	12.38 (14.63)^*^	0.20 (0.29)	3.35 (2.69)	3.18 (2.46)	3.58 (3.17)	4.70 (3.16)	3.10 (3.01)
	T24	0.50 (0.73)	2.40 (2.14)^*^	12.84 (12.63)^**^	0.15 (0.22)^**^	3.33 (2.19)^*^	3.67 (2,32)^*^	2.81 (2.32)^**^	2.81 (2.26)^*^	3.00 (2.59)^*^
Total patients with IBP, *n* = 75	T0	0.88 (1.08)	3.44 (2.45)	17.65 (20.64)	0.39 (0.49)	3.87 (2.99)	5.33 (2.87)	4.25 (3.05)	4.32 (2.18)	3.92 (3.40)
	T6	0.75 (1.08)	3.07 (2.59)	17.01 (21.26)	0,25 (0,38)	3.36 (2.83)	4.45 (2.82)	3.65 (2.95)	3.61 (2.97)	2.98 (2.91)
	T12	0.49 (0.79)	2.59 (2.67)	11.95 (14.30)^*^	0,16 (0,30)^**^	2.67 (2.38)^*^	3.40 (2.29)^**^	3.08 (2.61)	3.57 (2.77)	2.47 (2.54)
	T24	0.48 (0.75)	2.12 (2.13)^**^	10.73 (12.12)^**^	0,14 (0,29)^***^	2.25 (2.17)^**^	2.63 (2.24)^***^	2.26 (2.12)^**^	2.26 (2.14)^**^	2.28 (2.30)^**^

Data are expressed as mean ± SD. The test Kruskal Wallis repeated measures test and Dunn's multiple comparison test were used

*** *p < 0.0001 vs. T0*,

** *p < 0.001 vs. T0*,

* *p < 0.01 vs. T0*.

*BASMI, Bath Ankylosing Score Metrology Index; MASES, Maastricht Ankylosing Spondilities Enthesitis Score; BASFI, Bath Ankylosing Spondylitis Functional Index; HAQ: Health Assessment Questionnaire; BASG1, Bath Ankylosing Spondylitis Patient Global Score 1; BASG2, Bath Ankylosing Spondylitis Patient Global Score 2; VAS pain, Visual Analog Scale pain; VAS dis act, Visual Analog Scale Disease Activity; VAS pain N, Visual Analog Scale pain night*.

**Table 6:** Values of serological and disease activity indices from T0 to T24 in the whole group of patients (*n* = 75) and in three cohorts (axSpA imaging arm, axSpA clinical ± imaging arm, not full ASAS axSpA).

		**ESR**	**CRP**	**hs CRP**	**MMP3**	**IL-17**	**IL-22**	**IL-23**	**BASDAI**	**ASDAS**
axSpA imaging arm	T0	17.52 (12.98)	4.81 (3.61)	2.54 (2.79)	3.04 (2.69)	3 (3.12)	6.44 (3.52)	29.3 (31.2)	39.50 (25.99)	2.29 (0.86)
	T6	13.55(11.59)	3.04 (2.01)	2.34 (2.23)	3.54 (2.11)	3 (3.08)	6.12 (3.92)	28.5 (38.6)	29.17 (26.19)	2.14 (0.98)
	T12	15.06 (10.23)	4.06 (2.98)	2.26(3.21)	3.43 (2.80)	3 (2.89)	5.57 (5.23)	20(21.7)	29.06 (26.71)^*^	1.78 (0.87)^*^
	T24	15.13(5.73)	3.13 (1.31)	1.44 (1.28)	1.89(1.02) ^*^	3 (2.77)	5.1 (5.08)	20 (20.5)^*^	18.72 (18.25)^**^	1.06 (0.56)^**^
axSpA clinical ± imaging arm	T0	15.14 (11.76)	3.17 (3.32)	1.96 (1.61)	3.41 (3.35)	3 (3.19)	5.34 (2.71)	21.87 (5.21)	44.27 (25.03)	2.61 (0.56)
	T6	14.88 (9.27)	3.12 (2.19)	1.56 (1.71)	3.51 (3.12)	3 (3.11)	5.91 (2.45)	26.93 (6.46)	35.11 (24.70)	1.95 (0.79)
	T12	11.73 (9.39)^*^	4.05 (6.31)	2.92 (3.79)	2.12(1.82)	3 (3.03)	6.37 (1.63)	20 (21.2)	25.48 (17.78)^**^	1.59 (0.54)^*^
	T24	11.86 (5.54)	3.59 (3.84)	1.65 (2.22)	1.30 (1.50)	3 (2.99)	5 (0.11)	20 (20.9)	20.73 (18.76)^***^	1.33 (0.69)^***^
not full ASAS axSpA	T0	21.68 (21.19)	4.24 (3.28)	2.76 (3.76)	2.47 (2.68)	3 (3.97)	9.83 (8.54)	20.69 (21.56)	53.48 (24.97)	2.66 (0.88)
	T6	19.73 (17.01)	6.35 (10.33)	2.85 (3.91)	2.66 (2.74)	3 (3.16)	9.56 (8.28)	21.34 (20.17)	45.66 (27.37)	2.51 (1.19)
	T12	16.06 (13.06)	4.22 (3.67)	2.94 (3.50)	1.74 (1.71)	3 (3.12)	6.71 (5.46)	20.18 (19.78)	31.26 (19.67)^*^	2.02 (0.99)
	T24	18.25 (10.21)	3.63 (2.03)	1.50 (2.10)	1.11 (0.54)	3 (2.89)	5.31 (4.99)^*^	20.12 (15.65)	24.80 (19.67)^**^	1.34 (0.61)^*^
Total patients with IBP, *n* = 75	T0	17.99 (15.85)	3.98 (3.42)	2.11 (2.50)	3.01 (2.80)	3 (4.11)	12.58 (11.69)	21.53 (60.56)	46.01 (25.07)	2.54 (0.77)
	T6	16.09 (13.05)	4.29 (6.33)	2.75 (3.62)	2.84 (2.93)	3 (3.75)	10.43 (8.95)	21.79 (22.13)	36.90 (26.51)	2.19 (1.01)
	T12	14.48 (10.80)^*^	4.10 (4.62)	2.84 (3.27)	1.94 (1.91)	3 (3.10)	9.34 (5.84)	20.52 (19.87)	28.37 (21.05)^**^	1.79 (0.82)^**^
	T24	14.94 (7.30)	3.46 (2.75)	1.90 (2.18)	1.88 (1.56)	3 (3.84)	11.37 (6.12)	20.81 (15.31)	21.34 (18.07)^***^	1.34 (0.61)^***^

Data are expressed as mean ± SD. The test Kruskal Wallis repeated measures test and Dunn's multiple comparison test were used

*** *p < 0.0001 vs. T0*,

** *p < 0.001 vs. T0*,

* *p < 0.01 vs. T0*.

*ESR, erytrocyte sedimentation rate; CRP, C-reactive protein; hs-CRP, high sensitive C-reactive protein; MMP3 matrix-metallo-proteinase 3; IL, Interleukine; BASDAI: Bath Ankylosing Spondylitis Disease Activity Index; ASDAS, Ankylosing Spondylitis Disease Activity Score*.

## Discussion

Identifying novel biomarkers in rheumatic diseases will facilitate early detection and diagnosis of SpA and assist in the efforts to monitor disease activity and treatment response (7, 8). Currently, there are no established reliable diagnostic serum biomarkers assisting clinicians in identifying early stages of axSpA. New biomarkers have recently undergone extensive examination since HLA-B27, the biomarker commonly used in SpA, and CRP and the ESR, are often unable to assess disease activity (13–16). The CRP and ESR, recognized as acute-phase proteins reflecting systemic inflammation, are commonly utilized in clinical practice (22), although they do not reflect completely the inflammation process in axSpA due to the low sensitivity and specificity (23). An elevated CRP is one of the ASAS classification criteria for axSpA, while CRP level is taken into consideration in the ASDAS and correlates with the BASDAI (2). However, an elevated CRP or ESR is detected in only about 40–50% of AS patients (24). The degree of observed inflammation, in fact, fluctuates during the course of axSpA. In general, CRP level is higher in patients with r-axSpA vs. nr-axSpA (25). Although CRP is within the normal range in a large proportion of patients with active axSpA, it is still widely considered a reliable parameter of disease activity. In fact, CRP levels correlate moderately with MRI inflammation (10, 11, 15, 25). In addition, CRP has been found to be a reliable biomarker for monitoring treatment response and predicting further radiographic progression. Several studies have demonstrated that CRP levels drop significantly during anti-TNFα treatment (11, 13, 15). Modifications in CRP levels are correlated with changes in BASDAI and MRI scores, while elevated baseline CRP levels are associated with a good treatment response (13, 15) and represent a strong positive predictor of radiographic sacroiliitis progression, in particular for the progression from nr-axSpA to AS (15). Several prospective studies have also demonstrated that elevated CRP levels are independently associated with radiographic spinal progression in axSpA patients (26). In summary, CRP currently appears to be a reliable biomarker for assessing disease activity and predicting structural progression and treatment response. Conversely, ESR appears to be a non-specific measure of inflammation which may be influenced by a variety of other non-rheumatic conditions and comorbidities. However, some studies reported that higher ESR levels such as increased CRP levels are independently associated with structural disease progression in nr-axSpA patients (26). The current study investigated whether there were differences in ESR, CRP, and some biomarkers (MMP3, IL-17, IL-22, IL-23) in relation to clinical indices of disease activity and the presence/absence of sacroiliitis signs on X-rays and MRIs in an Italian cohort with early and confirmed axSpA. Although a significant difference was found in the three cohorts with regard to the prevalence of sacroiliitis on MRIs and X-rays and on the SPARCC SIJ score, we did not find any differences in clinical and disease activity indices. Higher indices were not found in the patients with active sacroiliitis on MRI compared to those without inflammatory changes in the SIJ or with initial signs of radiographic sacroiliitis. This finding may be attributable to both the early stages of axSpA and the small sample size. In fact, several studies (10–12, 14, 15, 27) reported higher values of clinical, functional, and disease activity indices in AS, in patients with long disease duration compared to subjects with nr-axSpA. We also uncovered a significant correlation at T0 between inter- and intra-clinical activity and disease activity indices; and between ESR and hs-CRP, as reported in literature (10, 11). The interesting correlation found between serological MMP3 levels and mSASSS, which is indicative of metalloproteinases' role in the bone formation process, was in line with previous findings by Maksymowych et al. (28) who reported that independently of other indices, serum MMP3 levels could predict 2-year radiographic progression in axSpA patients. Our data also showed that MMP3 correlates with other inflammatory, functional, and disease activity indices (such as CRP, BASFI, and BASDAI) and is increased in the serum of patients with active axSpA, in agreement with previous studies (28–33). It has been shown that MMP3 correlates with disease activity indices even in psoriatic arthritis (PsA) (34). As reported by Maksymowych et al. anti-TNF treatment induces a significant decrease in serum MMP3 levels as well as in ESR and BASDAI (28). van Kuijk et al. (35) studied 24 SpA patients randomized to receive adalimumab or placebo and found reduced serum MMP3 levels only 4 weeks after the onset with anti-TNFα therapy, while no change was noted in the serum levels of the placebo group. MMP3 was elevated in active forms of SpA or in cases presenting important clinical involvement, supporting the hypothesis that MMP3 might represent a reliable marker of disease activity. The correlation between mSASSS values and serum biomarkers should be evaluated in prospective studies. Recently, several studies have focused on the research and evaluation of ILs involved in the pathogenesis of SpA (28). IL-6 and IL-1 are the most widely studied proinflammatory cytokines (10, 36–38), produced by a variety of immune cells and implicated in the production of a series of acute phase proteins such as amyloid serum A and the CRP. These two mediators are involved in the initiation and maintenance of the inflammatory process by stimulating the migration and proliferation of neutrophils at the site of inflammation, and by regulating the activation and differentiation of T lymphocytes. Some recent studies have demonstrated that IL-1, IL-6, and other interleukins may play a role as inflammatory biomarkers (36–38). Among these, IL-17 and IL-23 are key cytokines in the Th17 pathway (39–49). A series of studies have reported that elevated levels of IL-17 and IL-23 are found in plasma and serum of AS patients, which are associated with a higher disease activity (50–53). IL-17 is mainly produced by Th17+ T-helper cells under the IL-23 stimuli. The number of IL-17+T cells and the IL-17 serum levels in circulation is higher in AS patients with respect to healthy individuals and to subjects with degenerative alterations (40–44, 54, 55). Interestingly, the number of IL-17+T cells is increased in patients with early active axSpA (55). IL-23 serum levels and expression in the subchondral bone marrow from the facet joints (43, 54–56) are up-regulated in AS patients. Few studies have found a positive correlation between IL-23 serum levels and CRP values, and MRI inflammation or clinical disease activity (42, 56). The IL-17/IL-23 axis is also crucially involved in other inflammatory and autoimmune diseases (39). Both IL-17 and IL-23 levels drop significantly in responders to anti-TNF α therapy while they rise in non-responders (42). Nowadays, both aforementioned cytokines have become important therapeutic targets (45–49). Clinical trials showed that ustekinumab—a monoclonal antibody against the p40 subunit shared by IL-12 and IL-23—and secukinumab—a monoclonal antibody against IL-17—yielded clinical improvements in axSpA patients (45–49). However, some studies failed to establish a correlation between systemic IL-17 levels with inflammatory indices or clinical disease activity parameters (42, 54, 56). In our study, we assessed whether there were significant differences in IL-17, IL-22 and IL-23 levels at T0 in the three cohorts of patients (*axSpA imaging arm, axSpA clinical* ± *imaging arm, not full ASAS axSpA*) and no difference was detected. Such findings may owe primarily to the very early stage of the disease (inflammatory LBP≥3 months and <2 years). It has also to be underlined that the inclusion criteria for this study were primarily the presence of inflammatory LBP and clinic/immunogenetic and imaging SpA features; the study population was divided in three cohorts only afterwards, following the characteristics evidenced on imaging (presence or absence of SIJ involvement in MRI and X-Rays and of spine involvement in MRI). Another possible explanation for the absence of elevated serum levels of inflammatory markers such as CRP and ESR may be that inflammation is restricted to specific tissue compartments and does not extend to the systemic circulation and/or lymphoid organs in axSpA. Accordingly, previous studies reported that the immupathology of affected tissues and inflammatory alterations were not always reflected in the peripheral blood compartment (57, 58). These findings are in line with previous studies (59, 60). Besides, in our population we did not find a significant correlation between IL-17 and IL-23 values, and other serological, clinical, and disease activity indices. Therefore, these biomarkers appear to be just indicative of the early stages of the disease, unlike IL-22 levels which correlated with some clinical indices of SpA such as BASFI, BASG1, HAQ, VAS pain (16). Elevated IL-22 levels have been reported in other forms of SpA—mostly PsA—and it correlates with clinical and disease activity indices suggesting an inflammatory role in the peripheral synovitis and in the diffuse skin psoriasis (61, 62). However, we found no significant correlation between skin involvement and serum levels of IL-22, because patients with psoriasis had a very limited skin involvement or only onicopathy with PASI <1. Some studies found higher IL-22 levels in axial SpA, thus suggesting a possible role in active sacroiliitis. However, we were not able to corroborate these findings in our study population which may be attributable to our small sample size (16). It would be interesting to evaluate if there is a correlation between the IL-22 levels and the presence/absence of active sacroiliitis in a larger sample size. In our study we also found an interesting correlation between ESR and clinical indices and mSASSS, although CRP currently appears to be a reliable biomarker for assessing disease activity and predicting structural progression and treatment response and ESR appears to be a non-specific measure of inflammation that may be influenced by a variety of other non-rheumatic conditions and comorbidities. In fact, some studies reported that higher ESR levels such as increased CRP levels are independently associated with structural disease radiographic spinal progression in nr-axSpA patients **[**26]. During the follow-up from T0 to T24, a significant reduction of functional and disease activity indices was observed in all patients: MASES (*p* = 0.008), BASG1 (*p* = 0.02), BASG2 (*p* < 0.0001), HAQ (*p* = 0.0002), VAS pain (*p* = 0.01), VAS pain night (*p* = 0.04), VAS disease activity (*p* = 0.05), BASFI (*p* = 0.02), BASDAI (*p* < 0.0001), ASDAS (*p* < 0.0001). On the other hand, BASMI, ESR, and CRP did not decrease significantly. There were no differences in the serological markers (ILs, MMP3, and hsCRP) values during the 2-year follow-up period in the three groups. Considering patients subdivided into the 3 cohorts, we observed a downward trend for all functional and disease activity indices, which in some cases was statistically significant, particularly in two cohorts (*axSpA imaging arm, axSpA clinical* ± *imaging arm*), though the decrease as comparable across all 3 cohorts. The improvement of the clinical, functional, and disease activity indices is probably due to the pharmacological treatment initiated after the diagnosis of axSpA; at the same time a reduction of inflammatory lesions on spinal and pelvis MRI was observed.

## Conclusions

The importance of early diagnosis and treatment of axSpA has been highlighted by many studies. Although the utility of some—serological markers in diagnosing AS and axSpA, in monitoring disease activity, and in predicting patients most at risk for poor outcome has been investigated by numerous researchers, ESR, and CRP have proven to be inadequate parameters for monitoring SpA disease activity (23, 59). Human studies related with axSpA and IL-17, IL-22, IL-23 are until now few. The strength of our study is based on the investigation as candidate biomarkers of ESR, hsCRP, and new molecules so far little studied (such as IL-17, IL-22, IL-23, and MMP3) in correlation with imaging and approved clinical indices in a very early stage of axSpA disease. In the current study in early axSpA patients with different types of axial involvement (presence or absence of radiographic sacroiliitis and of active sacroiliitis on pelvic MRI), out of all candidate biomarkers, none did change significantly over the study period even though there was a significant decrease in disease activity indices and imaging scores. ILs, MMP3, and hs-CRP values were not significantly higher in any of the cohorts and were not correlated with radiographic SIJ involvement. A significant correlation between IL-22 and some disease activity indices and between MMP3 and hs-CRP was instead noted. Our data suggest that the disease processes driving axSpA are not reflected in the alterations observed in the peripheral blood compartment. Some limitations of our study include: the small number of patients and the variety of treatments in each cohort, the early disease stage (LBP ≥3 months, ≤2 years, onset <45 years) and the absence of inclusion of LBP patients who were not axSpA. Although, it was claimed that some of the biomarkers correlated with some disease activity indices and with mSASSS, all correlations were rather weak. Further studies are warranted to assess in a more exhaustive manner the validity and reproducibility of the disease activity biomarkers considered here in identifying and monitoring the progression of axSpA.

## Data Availability

The datasets generated for this study are available on request to the corresponding author.

## Author Contributions

ML participated in drafting the manuscript as well as analyzing, acquiring, and interpreting the data. RR conceived and designed the study and participated in data processing and in drafting the manuscript. SV, VS, and CL took spine and pelvis X-rays, acquired MRI images and read them. MZ and CC carried out laboratory testing. AO, MFe, MFa, and PP participated in acquiring the data. All the authors have made substantial intellectual contributions to the study, have reviewed the manuscript, and have approved the version being submitted.

### Conflict of Interest Statement

The authors declare that the research was conducted in the absence of any commercial or financial relationships that could be construed as a potential conflict of interest.
